# Molecular Epidemiology and Clinical Features Analysis of Respiratory Adenovirus Infections Reveals Correlations between Genotype, Inflammatory Biomarkers, and Disease Severity

**DOI:** 10.1155/2020/4357910

**Published:** 2020-10-21

**Authors:** Hui Dai, Hongli Xi, Li Huang, Zhaohu Yuan, Yike Liao, Yongcai Tang, Jun Liao, Ling Min, Zhiwu Yu

**Affiliations:** ^1^Department of Laboratory Medicine, Guangzhou First People's Hospital, Guangzhou Medical University, Guangzhou, Guangdong 510180, China; ^2^Department of Laboratory Medicine, Guangzhou First People's Hospital, School of Medicine, South China University of Technology, Guangzhou, 510180 Guangdong, China; ^3^Division of Laboratory Science and Laboratory of Tumor and Virology, Affiliated Cancer Hospital & Institute of Guangzhou Medical University, Guangzhou, Guangdong 510095, China; ^4^Department of Blood Transfusion, Guangzhou First People's Hospital, School of Medicine, South China University of Technology, Guangzhou, 510180 Guangdong, China; ^5^Health Science Center, Peking University, Peking 100191, China

## Abstract

**Background:**

Human adenoviruses (HAdVs) are commonly causing respiratory disease. We molecularly genotyped HAdV circulating in Chinese hospitalized children with respiratory infections and summarized the clinical profiles and common inflammatory biomarkers, so as to better determine their associations with disease severity.

**Method:**

Children with respiratory single HAdV infection cases that occurred from December 2017 to March 2019 were enrolled for a cross-sectional study. Clinical/laboratory features based on the genotypes of respiratory HAdV infection were reviewed for comparative analysis.

**Results:**

A total of 84 patients were enrolled, and HAdV types were identified from 82 patients. Species B (HAdV-7, 44%; HAdV-3, 43%, and HAdV-14, 5%) was the most common, followed by C (HAdV-2, 4% and HAdV-1, 1%) and E (HAdV-4, 1%). Severe HAdV infection and HAdV-7 infection groups were associated with significantly longer duration of fever and hospitalized days, higher morbidity of tachypnea/dyspnea, more pleural effusion, more respiratory rales, more frequently required mechanical ventilation, and significantly higher fatality rate. The elevated procalcitonin (PCT) and C-reactive protein (CRP) levels were significantly associated with severe HAdV infection.

**Conclusions:**

HAdV-7 and HAdV-3 were the most common types among children with respiratory adenovirus infection; vaccines against these two genotypes are in urgent need. PCT and CRP are significantly associated with the severity of HAdV infection.

## 1. Introduction

Family Adenoviridae, genus Adenovirus, human adenoviruses (HAdVs) are highly contagious pathogens with marked differences in tissue tropism and clinical syndromes based on different species' infection involvement [1]. Up now, there are more than 90 genotypes of HAdV that divided into seven species from A to G based on bioinformatics analysis of genomic sequences and phylogenetic analyses [2]. Human adenoviruses (HAdVs) were the leading causative pathogen, usually responsible for 5%-7% of respiratory infections in infants and children [3]; the disease they cause are present with ranged from mild and self-limiting to severe pneumonia, even occasionally led to death [4]. Species B (mostly HAdV-3, -7, -14, and -55) and species E (HAdV-4) are commonly causing acute respiratory disease (ARD), including bronchopneumonia, community-acquired pneumonia (CAP), and upper respiratory tract infection [4–9], as well as outbreaks among crowded populations with the lack or lower level of herd immunity, such as infants and children [10–12].

However, differences in the disease severity to the various genotypes of HAdV, the association between clinical/laboratory profiles, and the circulation patterns of HAdV have not been well studied. Epidemiological surveillance of the current circulation patterns of HAdV in children may have the potential to prepare precautions in advance for an outbreak, since virus usually circulating in the normal population serve as a reservoir [13]. Links between the molecular epidemiology of HAdV and the inflammatory biomarkers, such as total white blood cell counts (WBC), serum C-reactive protein (CRP), and procalcitonin (PCT), may provide clues to clarifying the mechanisms underlying the disease severity of HAdV infections in children.

Here, we conducted a cross-sectional study based on the molecular epidemiology and clinical/laboratory features of febrile respiratory HAdV infections among children in our tertiary care pediatric department. Clinical and laboratory data were analyzed for comparing the differences between genotypes that belonged to species B. Comparison of the inflammatory biomarker levels between severe and nonsevere respiratory HAdV infection was also conducted in detail.

## 2. Materials and Methods

### 2.1. Study Design and Setting

This study enrolled hospitalized participants who were all later diagnosed with a respiratory single HAdV infection confirmed by real-time fluorescent polymerase chain reaction (PCR) between December 2017 and March 2019 in the pediatric department of Guangzhou First People's Hospital. Nasopharyngeal swabs collected from children under 14 years old with febrile respiratory infection symptoms admitted to the pediatric unit by trained personnel. Nasopharyngeal swabs were subjected to use the xTAG® Respiratory Viral Panel Fast (RVP FAST, Luminex, Canada) multiplex RT-PCR for detection of respiratory viruses, including influenza A and B viruses, human parainfluenza virus, respiratory syncytial viruses A and B (RSVA and RSVB), human coronaviruses, human metapneumovirus, human bocavirus (HBoV), rhinovirus, and human adenoviruses (HAdVs). This kit incorporates multiplex RT-PCR with Luminex's proprietary universal tag sorting system on the Luminex® xMAP 200IS platform. The assay amplifies specific fragments of the viral RNA or DNA via a single multiplex RT-PCR or via a PCR with hybridisation detection using specific capture probes [14].

The whole blood and serum specimens were collected from participants, according to the standard operating procedures within 24 hours after admission. Blood and sputum cultures were also performed, and serum samples were analyzed to detect *Mycoplasma pneumoniae* and *Chlamydia pneumoniae* antibodies. Cases diagnosed with concomitant bacterial infection or coinfected with other respiratory viruses were excluded. The patients were classified into mild pneumonia (nonsevere) group and severe group based on the clinical evidences.

### 2.2. Definition of Clinical Severity

Based on the clinical features, nonsevere cases in this study were defined by the presence of common pneumonia in chest radiographs, plus symptoms of dyspnea (lower chest wall indrawing), or tachypnea (respiratory rate > 30 breaths/min), and (or) little amount of pleural effusion that does not require respiratory support.

Severe cases were based on the presence of pneumonia with rapidly progressing lung shadow with multiple or single lobar/segment consolidation and (or) medium or massive pleural effusion, plus symptoms of respiratory rate > 50–60 breaths/min, or PaO2 < 70 mmHg and (or) SpO2 < 90% with respiratory distress and exhaustion that requiring either invasive or noninvasive respiratory support or illness resulting in death.

### 2.3. Ethical Approval

This study protocol performed in accordance with the ethical guidelines was approved by the Medical Ethics Committee of Guangzhou First People's Hospital, Guangzhou Medical University. Informed consents for the experimental protocols were obtained from all the participants or guardians of underaged enrolled children. Data records of the samples, sample collection, and analysis are deidentified and completely anonymous.

### 2.4. Identification of HAdV Molecular Types

Nucleic acids were extracted from the HAdV-positive nasopharyngeal swab and further molecular typed by PCR amplification, followed by Sanger sequencing of all seven hypervariable regions of the partial hexon gene. Molecular type was determined by Basic Local Alignment Search Tool-Nucleotide (BLASTN) of the assembling contigs for research on GenBank nucleotide database of existing sequences similar to the raw sequences that have been shown to successfully determine most HAdV genotypes [15].

### 2.5. Measurement of WBC, CRP, and PCT

The WBC of whole blood was detected using a routine automated flow cytometer Sysmex XE5000 (Sysmex, Tokyo, Japan); the sera CRP values were determined using the ADVIA 2400 automatic biochemical analyzer with matching reagents (Siemens, Berlin, Germany), and PCT concentrations were quantified using COBAS 411 automatic immune analyzer with matching reagents (Roche, Mannheim, Germany).

### 2.6. Statistical Analysis

The qualitative data were summarized as frequency and percentage, and the quantitative data were presented as mean ± standard deviation (mean ± SD) for normal distribution and median (range, extreme values at both ends) or median value with the interquartile range (IQR) for nonnormal distribution. Student's *t*-test, nonparametric Mann–Whitney *U* test, chi-squared test, or Fisher exact test were performed where appropriate among continuous variables and categorical variables between groups in the SPSS (version 19.0; Chicago, USA) program to determine the difference. Receiver operating characteristic (ROC) curve analysis was performed to evaluate the performance of inflammatory biomarkers (WBC, CRP, and PCT) to predict HAdV-7 or severe respiratory HAdV infection. The optimal cutoff value was determined by the Youden index that employing maximized the sum of sensitivity and specificity. A comparison between the area under the ROC curves (AUC) was performed utilizing a *Z*-test. *P* values of <0.05 were denoted to be statistically significant when a two-tailed test was employed.

## 3. Results

### 3.1. Frequency of the HAdV Species and Genotypes

**Figure 1:**
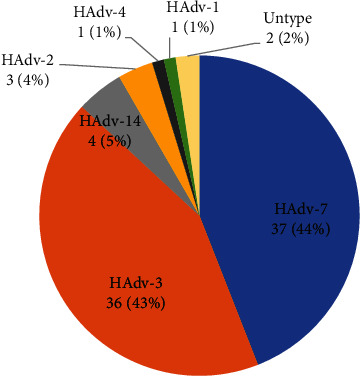
The frequency of HAdV species and genotypes identified among hospitalized children with respiratory infections.

Within the study period, 84 target children with single HAdV infection were enrolled, and 82 (97.6%) samples were identified for the HAdV species and genotypes, while 2 samples had an insufficient volume for further study. They included 3species and 6 types, i.e., species B (types 3, 7, and 14), species C (types 1 and 2), and species E (type 4). Species B was the most common (HAdV-3, 43%; HAdV-7, 44%; and HAdV-14, 5%), followed by species C (HAdV-1, 1% and HAdV-2, 4%) and species E (HAdV-4, 1%) (Figure 1).

### 3.2. Distribution of the HAdV Genotypes

**Figure 2:**
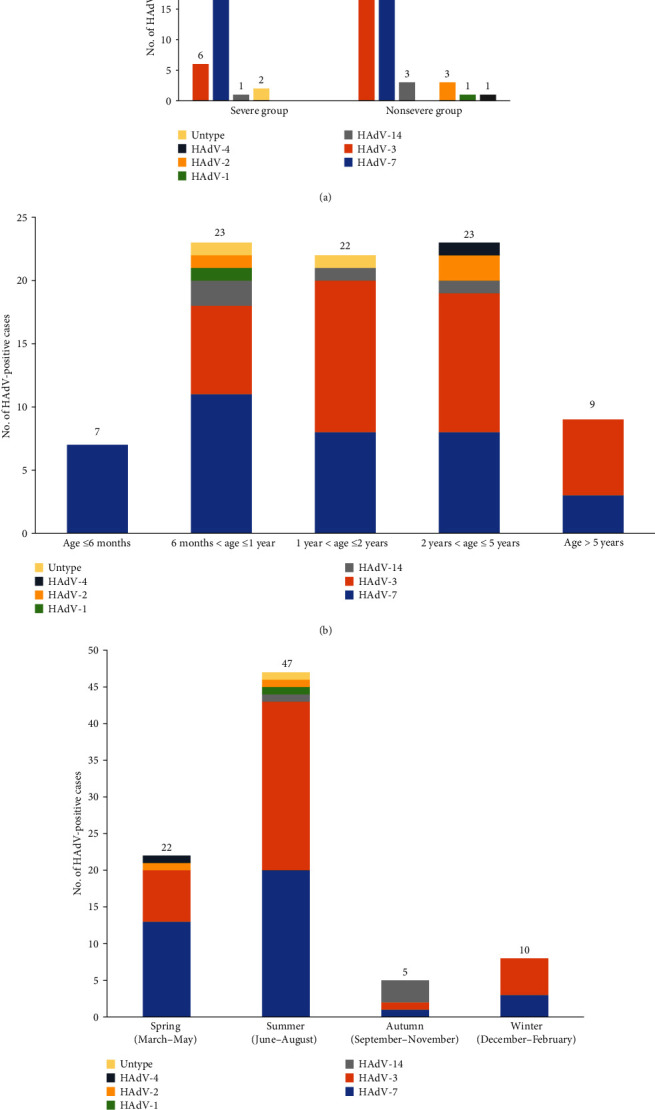
The distribution of HAdV genotypes according to severity groups (a), age groups (b), and seasons (c).

In this study, HAdV-positive cases account for febrile respiratory infection in hospitalized children in Southern China predominantly appeared in the summer; the genotypes of HAdV-7 and HAdV-3 were actively circulating all year round (Figure 2(c)). Besides, the genotype of HAdV-7 is causing febrile respiratory infection in every age group (Figure 2(b)). The distribution of HAdV genotypes in severity groups showed that HAdV-3, HAdV-7, and HAdV-14 infections commonly occurred in both groups, only 3 cases of HAdV-2, one case of HAdV-1, and one case of HAdV-4 rarely observed in nonsevere group (Figure 2(a)). The proportion of HAdV-7 infection in the severe group was significantly higher than nonsevere infected population (67.9% vs. 32.1%; *P* = 0.002).

### 3.3. Demographic Characteristics of the Patient

The median age of children with HAdV infection was 27.9 months (3.2–111 months); 88% were under 5 years. When compared regarding age, no significant difference was found between the HAdV-3 group and HAdV-7 group (*P* = 0.201). However, severe infection was significantly associated with a younger age than nonsevere infection (*P* = 0.001). The male-to-female ratio of the children infected with HAdV was 60/24; the gender distribution was comparable between the HAdV-3 group and HAdV-7 group (*P* = 0.463), as well as between the severe infection group and nonsevere group (*P* = 1.00) (Tables 1 and 2).

### 3.4. Comparison of Clinical Manifestations and Laboratory Findings

All the enrolled children with HAdV infection had a fever as their main clinical symptom in this study. The mean peak of the body temperature of the subjects was 39.8°C (range: 37.6-42°C), with no difference between the HAdV-3 group and HAdV-7 group (*P* = 0.796) and so was between the severe infection group and nonsevere group (*P* = 0.175). The median duration of fever was 8.8 days (range: 1–34 days) among children with respiratory HAdV infections. The duration of fever in patients infected with HAdV-7 was significantly longer than in those with HAdV-3 infection (11.1 vs. 6.6 days, *P* = 0.002), and patients with severe HAdV infection had a longer duration of fever (12.9 vs. 6.7 days, *P* < 0.001) than those with nonsevere infection. Besides fever, cough (82.1%) was also the most commonly seen clinical manifestation of HAdV-infected patients, as well as rhinorrhea (27.4%), which both were comparable between the HAdV-3 group and HAdV-7 group and so was between the severe infection group and nonsevere group.

However, HAdV-7 infection and severe HAdV infection were associated with significantly higher morbidity of tachypnea/dyspnea, more pleural effusion, and more respiratory rales in comparison with HAdV-3 infected patients and those in the nonsevere group, respectively. Moreover, the patients with HAdV-7 infection or severe HAdV infection had a longer duration of hospitalized days, more frequently required mechanical ventilation, and a significantly higher fatality rate than those with HAdV-3 infection or those in the nonsevere group, respectively. Of note, more frequent transfer to the intensive care unit significantly associated with the severe HAdV infection group than the nonsevere group, without significant difference between the HAdV-3 group and HAdV-7 group (Tables 1 and 2). However, four (5%) inpatients infected with HAdV-7 died after high frequency oscillatory (HFOV) and extracorporeal membrane oxygenation (ECMO) treatment; all HAdV-3 infected children survived. All the deaths were associated with acute respiratory distress syndrome and had not major underlying diseases, except one premature child. The median duration from disease onset to death was 19 days (range from 13 to 25 days). The median duration of mechanical ventilator acquired was 9 days (range from 5 to 13 days), while the median duration of inotropic agent use was 3 days.

Serum CRP, PCT, and blood WBC were determined in this study. Of the 84 children with febrile respiratory infection due to HAdVs, 25% had increased (>15000/mm^3^) WBC count, and 31% had elevated (>40 mg/l) CRP levels. On the other hand, PCT levels of more than 0.05 ng/ml were detected in 78 (92.8%) and levels more than 0.5 ng/ml in 42 of 84 patients. The children with HAdV-7 infection had higher PCT values (3.34 vs. 0.74 ng/ml, *P* < 0.001) and a higher proportion of PCT elevated (>0.5 ng/ml) levels (72.9% vs. 27.8%, *P* < 0.001), when compared with children infected by HAdV-3. Meanwhile, the severe HAdV infection children had higher PCT values (4.39 vs. 0.77 ng/ml, *P* < 0.001) and a higher proportion of elevated (>0.5 ng/ml) PCT levels than those in nonsevere infection group (85.7% vs. 32.2%, *P* < 0.001). Although no significant difference was observed on elevated CRP levels between the HAdV-7 group and HAdV-3 group, the higher CRP level was significantly associated with the severe HAdV infection group in comparison to the nonsevere group (47.9 vs. 29 mg/l, *P* = 0.032). On the contrary, most blood WBC counts were within the normal range and did not significantly differ between the severe infection group and the nonsevere group. The children with HAdV-7 infection had a lower WBC (8900 vs. 13700/mm^3^, *P* < 0.001) and a lower proportion of increased (>15000/mm^3^) WBC (13.5% vs. 33.3%, *P* = 0.045) than those with HAdV-3 infection (Tables 1 and 2).

### 3.5. Predicting the Performance of the PCT, CRP, and WBC

**Figure 3:**
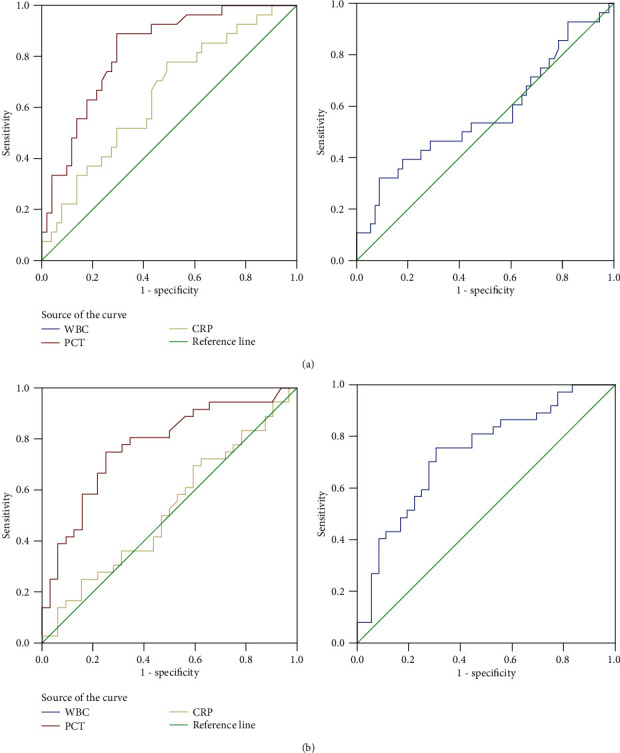
The ROC curves showing the ability of the PCT, CRP, and WBC to distinguish severe HAdV infection from nonsevere HAdV infection (a). The AUC value of PCT is significantly higher than of both CRP (*P* = 0.029) and WBC (*P* = 0.003). The ROC curves showing the ability of the PCT, CRP, and WBC to distinguish HAdV-7 infection from HAdV-3 infection (b). The AUC value of CRP is significantly lower than of both PCT (*P* = 0.0056) and WBC (*P* = 0.017). AUC: area under the curve; PCT: procalcitonin; CRP: C-reactive protein; WBC: whole blood cell; HAdV: human adenovirus.

The ROC curve was used to assess the predicting performance of the PCT, CRP, and the WBC for severe HAdV infection, which illustrated in Figure 3(a). PCT (AUC 0.822, 95% confidence interval, 0.729–0.915) had a significantly higher AUC value than of both CRP (AUC 0.647, 95% confidence interval, 0.521–0.774; *P* = 0.029) and WBC (AUC 0.572, 95% confidence interval, 0.434–0.710; *P* = 0.003) for distinguishing severe HAdV infection from nonsevere infection. The optimal cutoff values of PCT, CRP, and WBC, along with the Youden index, sensitivity, specificity, positive likelihood ratio, and negative likelihood ratio of the PCT, CRP, and WBC, are listed in Table 3.

The AUC values of PCT, CRP, and WBC on the ROC curve were also used to evaluate the predicting performance for HAdV-7 infection, which illustrated in Figure 3(b). CRP (AUC 0.520, 95% confidence interval, 0.382–0.659) had a significantly lower AUC value than of both PCT (AUC 0.773, 95% confidence interval, 0.661–0.886; *P* = 0.0056) and WBC (AUC 0.739, 95% confidence interval, 0.624–0.853; *P* = 0.017) for distinguishing HAdV-7 from HAdV-3 infection. The optimal cutoff values of PCT, CRP, and WBC, along with the Youden index, sensitivity, specificity, positive likelihood ratio, and negative likelihood ratio of the PCT, CRP, and WBC, are listed in Table 4.

## 4. Discussions

In this study, we determined the epidemiology and clinical/laboratory features of respiratory HAdV infections in otherwise healthy children without any underlying disease. We particularly observed the clinical/laboratory features associated with different HAdV types, especially the difference between HAdV-3 and HAdV-7, which have not been fully studied. Moreover, the associations between WBC, CRP, and PCT levels and the disease severity of respiratory HAdV infections were also the key issue to be well studied.

During this study period, we found that HAdVs were circulating all year round and predominantly appeared in summer in Guangzhou, Southern China. Species B was the most common circulating pattern (HAdV-3, 43%; HAdV-7, 44%; and HAdV-14, 5%) related to children with febrile respiratory infections; species E (HAdV-4) occurred sporadically. Most children aged <5 years were infected by HAdVs, and HAdV-7 affected every age group in our study. At the same time, HAdV-7 was mainly account for the severe respiratory infection. Our present results were in good accordance with those earlier epidemiological findings [8, 16–18].

Given the comparative analysis, fever and cough were the most common respiratory symptoms in children with HAdV infection, and most children (74, 88%) were under 5 years old; the children in the severe infection group were younger than those in the nonsevere group (16.1 vs. 33.7 months, *P* < 0.001), which indicated young age may be the risk factor for severe HAdV infection. Children with HAdV-7 infection have longer duration of fever and hospitalized days, higher morbidity of tachypnea/dyspnea, pleural effusion and respiratory rales, along with a higher occurrence of intensive care required, mechanical ventilation, and death than those with HAdV-3 infection, which was compatible with the clinical manifestations of the severe HAdV infection in this research. These results indicated that HAdV-7 had caused more severe respiratory infections and adverse outcomes than HAdV-3 in children, which also confirmed by recent similarity studies [7, 19]. No significant differences were found in terms of gender distribution and peak body temperature, as well as the occurrence of fever, cough, and rhinorrhea, between the HAdV-3 and HAdV-7 groups, and so were between the nonsevere and severe infection groups. Although all the HAdV-3 infected patients survived in this study, genotype of HAdV-3 is also important pathogenic in Chinese children, which account for high morbidity of adenoviral respiratory diseases and even death [8, 20]. The genotype of HAdV-3 is also needed to be aware of the impact on children's health.

Unlike other documented respiratory viral infections had low WBC and CRP levels, human adenovirus infection typically results in high WBC and CRP levels [21, 22], and the WBC and CRP levels had been compared between species B, C, and E in earlier studies [23–25]. In addition, the PCT value associated with changes in children with respiratory HAdV infection was observed in another study [26]. However, the associations between the WBC, CRP, and PCT values and adenovirus types have not been well understood; the differences in the WBC, CRP, and PCT levels to the various types and disease severity of respiratory HAdV infections have also not been fully explored. Strong inflammatory responses as evidenced by elevation of PCT with high WBC and CRP levels were mainly observed among children with human adenovirus species B infection in this study. Besides, we observed the PCT had the best predicting performance for severe HAdV infection and HAdV-7 infection. Usually, the normal level of PCT in noninfected persons is under 0.05 ng/ml, and an increase to 0.5 ng/ml or greater has been proposed to distinguish between bacterial and viral infections [27, 28]. However, more than 90% HAdV infection cases in the present study were found with elevated PCT (>0.05 ng/ml) levels, and half of the children had PCT > 0.5 ng/ml, higher PCT value, and higher proportion of elevated PCT (>0.5 ng/ml) levels that were significantly associated with severe HAdV infections and HAdV-7 infections. Thus, elevated PCT levels have been proposed to distinguish between bacterial and viral infections that were controversial, for more than 85% severe HAdV infections, and more than 70% HAdV-7 infections in the present study had elevated PCT (>0.5 ng/ml) levels. These findings suggest that low PCT levels support to guide withholding antibiotic therapy, and high PCT levels could not be proposed as bacterial infection. It may be involved with severe HAdV infection or HAdV-7 infection if the clinical examination is uncertain to diagnose a bacterial infection. In the meantime, we found that a higher CRP level was also significantly associated with the severe HAdV infection group, which looks like CRP levels in H1N1 influenza patients who develop a severe disease outcome [29]. Early reports showed that CRP could be abnormally elevated after adenovirus infection and was not associated with secondary infection, and the elevated level of CRP after adenovirus infection is significantly higher than influenza virus and respiratory syncytial virus infection [30, 31]. These findings suggested that CRP should be proposed as a biomarker of severe HAdV infection, although inflammatory events leading to increased CRP level in other respiratory virus pneumonia are incompletely clear. More respiratory viruses should be included to further assess CRP utility as a biomarker in predicting the severity of the respiratory viral infection. In our study, WBC counts in children with HAdV-3 showed significantly higher than those with HAdV-7 infections, though most WBC counts were within the normal range and did not significantly differ between the severe and nonsevere infection groups. This result may explain why HAdV-7 caused severe consequences than HAdV-3, as higher inflammatory markers may indicate higher innate immune responses leading to protective acquired immunity [32].

This study has a few limitations. First, the study population was enrolled at a single center as a cross-sectional study. Second, the study enrolled a relatively small number of patients. Further multicenter population-based studies are needed to address the selection bias. Third, we did not detect the viral load and the HAdV in blood to confirm the exact role of virus in clinical pathogenicity of severity, particularly those cases coinfection with other respiratory viruses should be further elucidated necessarily in future.

## 5. Conclusions

In conclusion, we have confirmed that HAdV species B, particularly HAdV-7 and HAdV-3, were the most common types among children with respiratory adenovirus infection during this study period. The vaccines against HAdV-7 and HAdV-3 are in urgent need in China, which may help in prevention of HAdV outbreaks. The elevated PCT values are significantly associated with severe HAdV infection or HAdV-7 infection; elevated PCT values as a first-line marker for the initiation of antibiotic therapy would deserve reconsideration. Moreover, the PCT and CRP are significantly associated with the severity of HAdV respiratory infections in children.

## Figures and Tables

**Table 1:** Demographics and clinical data of hospitalized children with single HAdV-3 or single HAdV-7 infection.

Characteristic	All patients (*N* = 73)		
	HAdV-3 (*N* = 36)	HAdV-7 (*N* = 37)	*P*
Male sex	25 (69.4)	29 (78.4)	0.463
Median age, month (range)	33.1 (6.7-92.5)	25.2 (3.2-111)	0.201
Fever duration days (range)	6.6 (1-21)	11.1 (2-34)	0.002
Median peak temperature, °C	39.8 ± 0.8	39.7 ± 0.5	0.796
Respiratory symptoms			
Cough	29 (80.6)	32 (86.5)	0.494
Rhinorrhea	11 (30.6)	7 (18.9)	0.249
Tachypnea/dyspnea	10 (27.8)	21 (72.4)	0.012
Pleural effusion	1 (2.8)	16 (43.2)	<0.001
Rales	19 (52.8)	31 (83.8)	0.004
Laboratory tests			
WBC, ×10^9^ cells/l	13.7 ± 6.3	8.9 ± 4.4	<0.001
>15 × 10^9^ cells/l	12 (33.3)	5 (13.5)	0.045
CRP, mg/l (IQR)	32.8 (54.7–5.1)	34.6 (59.6–8.1)	0.839
>0.15 mg/l	35 (97.2)	37 (100)	0.307
>40 mg/l	10 (27.8)	11 (29.7)	0.854
PCT, ng/ml (IQR)	0.74 (0.70-0.11)	3.34 (3.95–0.34)	<0.001
>0.05 ng/ml	32 (88.9)	36 (97.3)	0.199
>0.5 ng/ml	10 (27.8)	27 (72.9)	<0.001
Outcomes			
Hospitalized duration days (range)	10.7 (3-44)	17.7 (3-84)	0.01
Intensive care required	19 (52.8)	23 (62.2)	0.417
Mechanical ventilation	4 (11.1)	11 (29.7)	0.049
Death	None	4 (10.8)	<0.001

Values are no. (%) of patients or mean ± SD, unless otherwise indicated. Range: extreme values at both ends; IQR: interquartile range; WBC: whole blood cell; CRP: C-reactive protein; PCT: procalcitonin.

**Table 2:** Demographics and clinical features of hospitalized children with adenovirus infection.

Characteristic	All patients (*N* = 84)		
	Nonsevere infection (*N* = 56)	Severe infection (*N* = 28)	*P*
Male sex	40 (71.4)	20 (71.4)	1.00
Median age, month (range)	33.7 (4.8-111)	16.1 (3.2-58.7)	0.001
Fever duration days (range)	6.7 (1-21)	12.9 (3-34)	<0.001
Median peak temperature, °C	39.7 ± 0.7	39.9 ± 0.5	0.175
Respiratory symptoms			
Cough	43 (76.8)	26 (92.8)	0.07
Rhinorrhea	18 (32.1)	5 (17.8)	0.166
Tachypnea/dyspnea	12 (21.4)	22 (78.6)	<0.001
Pleural effusion	3 (5.3)	15 (53.6)	<0.001
Rales	29 (51.8)	28 (100)	<0.001
Laboratory tests			
WBC, ×10^9^ cells/l	12.4 ± 6.4	10.8 ± 6.5	0.307
>15 × 10^9^ cells/l	15 (26.8)	6 (21.4)	0.593
CRP, mg/l (IQR)	29 (44.3–4.6)	47.9 (63-18)	0.032
>0.15 mg/l	54 (96.4)	28 (100)	0.311
>40 mg/l	14 (25)	12 (42.8)	0.095
PCT, ng/ml (IQR)	0.77 (0.72–0.11)	4.39 (4.07–0.87)	<0.001
>0.05 ng/ml	51 (91.1)	27 (96.4)	0.658
>0.5 ng/ml	18 (32.2)	24 (85.7)	<0.001
Outcomes			
Hospitalized duration days (range)	10.2 (3 - 36)	20.2 (3 - 84)	<0.001
Intensive care required	21 (37.5)	26 (92.8)	<0.001
Mechanical ventilation	None	15 (53.6)	<0.001
Death	None	4 (14.3)	<0.001

Values are no. (%) of patients or mean ± SD, unless otherwise indicated. Range: extreme values at both ends; IQR: interquartile range; WBC: whole blood cell; CRP: C-reactive protein; PCT: procalcitonin.

**Table 3:** Performance of biomarkers for distinguishing severe HAdV infection from nonsevere infection.

	AUC	CI 95%	*P*	Optimal cutoff value	Sensitivity	Specificity	Positive likelihood ratio	Negative likelihood ratio	Youden index
PCT	0.822	0.729-0.915	< 0.001	0.755	88.90%	70.60%	3.02	0.157	0.595
CRP	0.647	0.521-0.774	0.033	18.05	77.80%	50.90%	1.59	0.436	0.287
WBC	0.572	0.434-0.710	0.286	5.945	32.10%	91.00%	3.6	0.745	0.232

AUC: area under the curve; CI: confidence interval; WBC: whole blood cell; CRP: C-reactive protein; PCT: procalcitonin.

**Table 4:** Performance of biomarkers for distinguishing HAdV-7 from HAdV-3 infection.

	AUC	CI 95%	*P*	Optimal cutoff value	Sensitivity	Specificity	Positive likelihood ratio	Negative likelihood ratio	Youden index
PCT	0.773	0.661-0.886	< 0.001	0.755	72.20%	75.00%	2.89	0.37	0.472
CRP	0.52	0.382-0.659	0.773	14.35	69.40%	40.60%	1.17	0.75	0.1
WBC	0.739	0.624-0.853	< 0.001	11.34	75.70%	69.40%	2.48	0.35	0.451

AUC: area under the curve; CI: confidence interval; WBC: whole blood cell; CRP: C-reactive protein; PCT: procalcitonin.

## Data Availability

The data used to support the findings of this study are available from the corresponding author upon request.
